# Phytochemical Diversity, Mechanistic Pharmacology, and Therapeutic Potential of *Alpinia oxyphylla*

**DOI:** 10.3390/foods15071212

**Published:** 2026-04-02

**Authors:** Taixia Chen, Shahidin, Qiangqiang Zhu, Yan Wang, Yilong Wu, Xiaoyun Wu, Wenjuan Yuan, Jun Sheng, Chengting Zi

**Affiliations:** 1 College of Food Science and Technology, Yunnan Agricultural University, Kunming 650201, China; 15870148875@163.com (T.C.); shahidinwazir998@gmail.com (S.); tianjiao125@126.com (Q.Z.); 2 Institute of Biofabrication Research, College of Science, Yunnan Agricultural University, Kunming 650201, China; wuxiaoyun79@gmail.com (X.W.); yuanwj0805@126.com (W.Y.); 3 Research Center for Agricultural Chemistry, College of Science, Yunnan Agricultural University, Kunming 650201, China; wangyan2882@163.com (Y.W.); ynau_wuyl@126.com (Y.W.); 4 Jiangsu Co-Innovation Center for Modern Production Technology, College of Animal Science and Technology, Yangzhou University, Yangzhou 225009, China; 5 Key Laboratory of Development and Utilization of Food and Medicinal Resources, Yunnan Agricultural University, Kunming 650201, China

**Keywords:** *Alpinia oxyphylla*, bioactive compounds, nutraceuticals, pharmacological activities, functional food applications

## Abstract

*Alpinia oxyphylla* Miquel is a perennial medicinal plant widely cultivated in the provinces of Fujian, Guangdong, and Hainan in China. The dried mature fruit of *A. oxyphylla*, officially recorded as Alpiniae Oxyphyllae Fructus in the pharmacopoeia of the People’s Republic of China (since 2012), is one of the four primary southern medicinal materials in traditional Chinese medicine (TCM). In TCM, the fruit is traditionally used to support kidney function, regulate urination, and alleviate gastrointestinal disorders such as diarrhea. Its continued use across Southeast Asia underscores its enduring ethnopharmacological relevance. The plant is rich in bioactive constituents, including terpenoids, flavonoids, diphenylheptanes, and sterols, which exhibit diverse biological activities, including antioxidant, anti-inflammatory, anticancer, neuroprotective, and gastrointestinal protective effects. Information on *Alpinia oxyphylla* was collected from multiple databases, including Web of Science, Google Scholar, PubMed, Baidu Scholar, ScienceDirect, CNKI, and the Pharmacopoeia of the People’s Republic of China. The search strategy included keywords related to *A. oxyphylla*, its chemical constituents, biological activities, pharmacological effects, traditional medicinal uses, and safety. A bibliometric analysis of 217 English-language publications (2014–2025) using CiteSpace revealed a marked increase in global research interest, with keyword clustering and burst analyses highlighting oxidative stress, Alzheimer’s disease, and cognitive enhancement as emerging research hotspots. Moreover, 692 patents were identified, demonstrating substantial technological innovation related to *A. oxyphylla*, particularly in essential oil formulations, functional foods, and health-promoting applications. Overall, this review integrates phytochemical, pharmacological, bibliometric, and patent perspectives to provide a holistic understanding of *A. oxyphylla* and its medicinal fruit, offering a solid scientific foundation for future research, standardization, and translational development.

## 1. Introduction

In recent years, increasing attention has been given to the comprehensive utilization of medicinal plant resources, as many species contain valuable bioactive components beyond their traditionally used parts [1]. *A. oxyphylla* is a perennial herb of the Zingiberaceae family. Its dried mature fruit, *Alpiniae oxyphyllae* Fructus (AOF), is the officially recognized medicinal material in traditional Chinese medicine and has been used for more than 1700 years, with the seeds constituting the primary medicinal part [2]. The species has a long history of medicinal use, dating back more than 1200 years. The earliest records, found in Materia Medica Gleanings, mention its presence in the Kunlun and Jiaotong regions, now part of the Lingnan area in southern China (https://cisema.com/en/chinese-pharmacopoeia-2020, (25 October 2025)). *A. oxyphylla* is mainly distributed in the southern provinces of China, including Hainan, Guangdong, Guangxi, and Fujian, where it thrives in warm, humid, and shaded forest environments. Among these regions, Hainan is recognized as the principal production area, and its fruits are regarded as the highest quality for medicinal use [3,4]. In particular, the Hainan variety, renowned for its superior medicinal properties, is also widely consumed as a health-care food, incorporated into vegetables, preserved fruits, beverages, and dietary supplements [5]. It has become an essential industrial crop in southern China due to its medicinal applications; however, its broader economic potential remains underexplored, as current use primarily focuses on fruit extracts for traditional Chinese medicine.

*A. oxyphylla* contains various chemical constituents, including essential oils, flavones, diarylheptanoids, sesquiterpenes, steroids, glycosides, fatty acids, and polysaccharides [6,7]. These constituents help alleviate oxidative stress by scavenging reactive oxygen species (ROS) and enhancing the activities of key antioxidant enzymes such as glutathione peroxidase (GSH-Px), superoxide dismutase (SOD), and catalase (CAT), as well as other endogenous antioxidant substances [8]. Currently, antioxidants are widely applied not only as food additives, health care products, and cosmetic ingredients but also as feed supplements and industrial stabilizers. Pharmacological studies have demonstrated that extracts of *A. oxyphylla* have potent antioxidant [1], anti-inflammatory [4], neuroprotective [9,10], urinary [11,12], and gastrointestinal [13] properties. It has also been reported to possess anticancer [11], antiaging [12], anti-Alzheimer’s disease [13], and immunomodulatory activities [14]. The 2020 edition of the Chinese Pharmacopoeia classifies *A. oxyphylla* as a warm and pungent herb that acts on the spleen and kidney meridians. It is traditionally used to warm the kidneys, strengthen the essence, reduce excessive urination, and alleviate diarrhea [15].

Although numerous studies have investigated the chemical constituents and pharmacological properties of *A. oxyphylla*, most findings remain fragmented, focusing on isolated aspects, such as individual metabolites or specific pharmacological activities. There is still a lack of systematic integration of traditional knowledge with modern pharmacological and molecular evidence. Moreover, despite the growing use of *A. oxyphylla* in conventional medicine and functional food, comprehensive evaluations of its bioactive constituents and therapeutic mechanisms are limited. Given the recent surge in publications concerning *A. oxyphylla*, a critical synthesis and bibliometric assessment of current research trends is essential to identify progress, challenges, and future directions in this field.

## 2. Methodology

### 2.1. Data Source Collection

The Web of Science (WoS) database is widely regarded as a key source for scientific research and bibliometric analysis [16]. In this study, data were retrieved from the Science Citation Index Expanded (SCI-EXPANDED) for the period 2014 to the present. The search strategy employed the keywords *Alpinia oxyphylla* Miq and *Alpiniae Oxyphyllae* Fructus to search titles, abstracts, and author keywords. The document types were restricted to English-language publications classified as articles, theses, and review articles. A total of 217 records published between 2014 and 2025 were identified in WoS and included in the analysis.

### 2.2. Data Analysis

This study used CiteSpace, a visual bibliometric analysis software developed by Professor Chaomei Chen [17]. CiteSpace was used to examine annual publication trends, collaboration networks, co-citation networks, keyword clustering, and keyword burst detection. As an advanced tool integrating systematic mapping, scientometrics, and visual analytics, CiteSpace enables the identification of research hotspots and emerging academic trends [16]. In the Web of Science database, the search was conducted using the keywords “*Alpinia oxyphylla*” and “*Alpinia oxyphylla* miq”. The time span was set from 2014 to 2025, with the document type limited to “Article” and the indexes restricted to SCI and SSCI. A total of 217 records were retrieved and exported in plain text format. The exported data were then imported into CiteSpace for conversion, during which duplicate records were removed, resulting in 217 valid articles for analysis. For the bibliometric analysis, the converted data were imported into CiteSpace with “WOS” selected as the data source, while the remaining parameters were kept at their default settings. The analyses were performed by selecting the appropriate node types in the “Node Types” panel, followed by visualization and refinement of the generated figures. Its primary purpose is to reveal the structural characteristics and evolutionary trajectories of a research field. All analyses in this study were performed using the latest version of CiteSpace (6.3). R1 (64-bit).

## 3. Bibliometric Analysis

### 3.1. Annual Publication Volume

**Figure 1:**
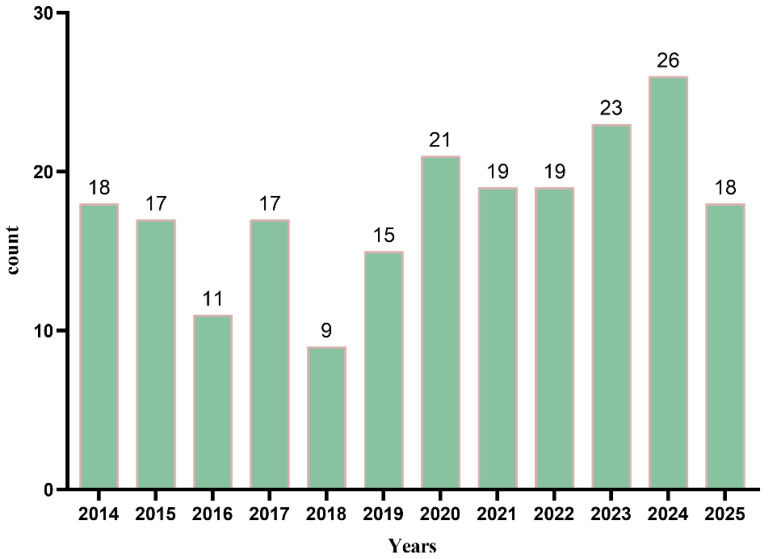
Annual publication volume of the English-language literature on *A. oxyphylla* research.

Analysis of the 217 records retrieved from the WoS Core Collection (WOSCC) shows that from 2014 to 2019, the number of publications remained relatively stable, with only minor fluctuations in 2016 and 2018. Beginning in 2020, however, the annual number of English-language publications increased steadily, reaching a peak in 2024 (Figure 1). This upward trend indicates growing international interest in *A. oxyphylla* research, which may be partly driven by heightened global attention to health and natural medicinal resources following the 2020 pandemic [18]. The increase in English-language output also reflects the expanding global engagement with *A. oxyphylla*, as researchers increasingly choose international journals to enhance academic visibility and facilitate cross-border collaboration. This trend is further reinforced by the comprehensive indexing of English-language databases such as WoS and the influential role of SCI/SSCI journals in research evaluation systems.

### 3.2. Country Analysis

A bibliometric analysis of the WoS database identified the top nine countries and regions contributing to *A. oxyphylla*-related research (Table 1). China ranked first with 186 publications, followed by South Korea with 19 publications. In particular, China and the United States lead in research output, creating a pronounced gap with other countries. This dominant position reflects the strategic prioritization and sustained research investment in *A. oxyphylla* by these nations.

The international collaboration network further illustrates the global landscape of scientific cooperation in this field. In Figure S1, the size of each node represents the volume of publications from each country, while connecting lines indicate active collaborative relationships. China, the United States, and South Korea exhibit particularly dense collaboration networks, highlighting their strong capacity for international research cooperation within the *A. oxyphylla* research domain.

### 3.3. Keyword Analysis

#### 3.3.1. Keyword Co-Occurrence Analysis

**Figure 2:**
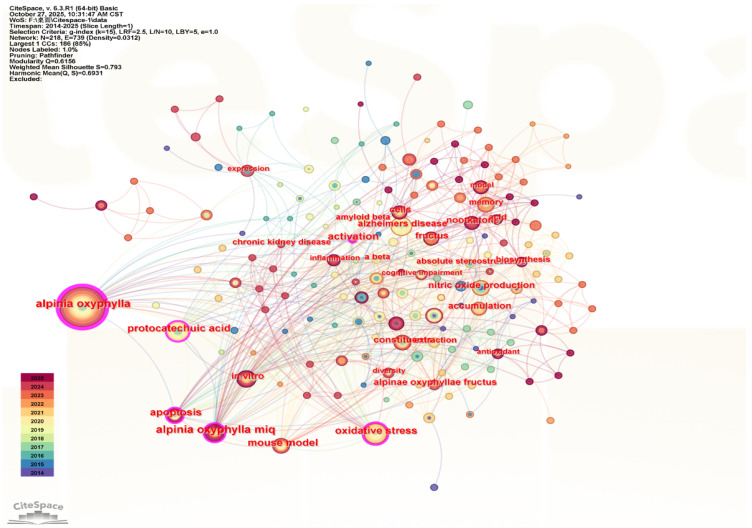
Keyword co-occurrence network of research on essential oils.

Keyword co-occurrence analysis can effectively reveal research hotspots within a specific field [19]. High-frequency, high-centrality keywords generally indicate popular or emerging research topics [20]. After excluding search terms such as *Alpinia oxyphylla* and *Alpinia oxyphylla* Miq., the most frequently occurring keywords, as shown in Table 2, include oxidative stress, protocatechuic acid, vitamin, Alzheimer’s disease, and nitric oxide production. Keywords with centrality values greater than 0.1 were considered to have a strong influence within the network. These keywords, oxidative stress (centrality = 0.14), protocatechuic acid (centrality = 0.16), apoptosis (centrality = 0.11), and activation (centrality = 0.15), represent major research hotspots in this field. A keyword co-occurrence network was generated, in which each node represents a keyword, the size of the node indicates its frequency, different colors indicate the year of the keyword’s appearance, and the connecting lines represent co-occurrence relationships between keywords (Figure 2).

#### 3.3.2. Keyword Cluster Timeline Analysis

**Figure 3:**
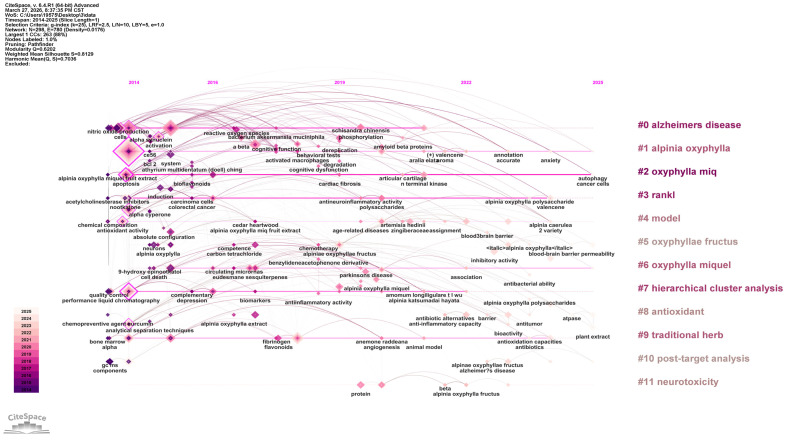
Keyword timeline graph.

Keyword clustering groups terms with similar or related topics to reveal the thematic structure of research within a field. The modularity value Q (Q > 0.3 indicates a significant clustering structure) and the mean silhouette value S (S > 0.5 indicates reasonable clustering; S > 0.7 indicates efficient and convincing clustering) are used as evaluation indicators. In this study, K-core clustering was applied for the analysis. A total of 11 clusters were identified in the literature, with Q = 0.6202 (>0.3) and S = 0.8129 (>0.7), demonstrating that the clustering structure is both significant and reliable. The representative clusters include: #0 alzheimers disease, #1 alpinia oxyphylla, #2 oxyphylla miq, #3 rankl#4 model, #5 oxyphyllae fructus, #6 oxyphylla miquel, #7 hierarchical cluster analys, #8 antioxidant, #9 traditional herb,#10 post-target analysis and #11 neurotoxicity (Figure 3). The emergence of these clusters reflects current trends in *A. oxyphylla* research. For example, clusters related to the bioactive constituents of the nut (alzheimers disease #0 and antioxidant #8) suggest a strong interest in these compounds due to their notable pharmacological activities. The cluster alpinia oxyphylla (#1) and traditional herb (#9) further underscores researchers’ emphasis on the fruit, suggesting potentially superior bioavailability or pharmacological activity. Clusters associated with functional activities, including post-target analysis (10) and neurotoxicity (#11), suggest that recent research has increasingly focused on elucidating the mechanisms underlying *A. oxyphylla*’s pharmacological effects. Clusters without meaningful grouping imply that some research areas related to *A. oxyphylla* remain limited and are still in early stages of exploration. These gaps provide a theoretical basis and opportunities for future studies on *A. oxyphylla*.

#### 3.3.3. Burst Analysis

**Figure 4:**
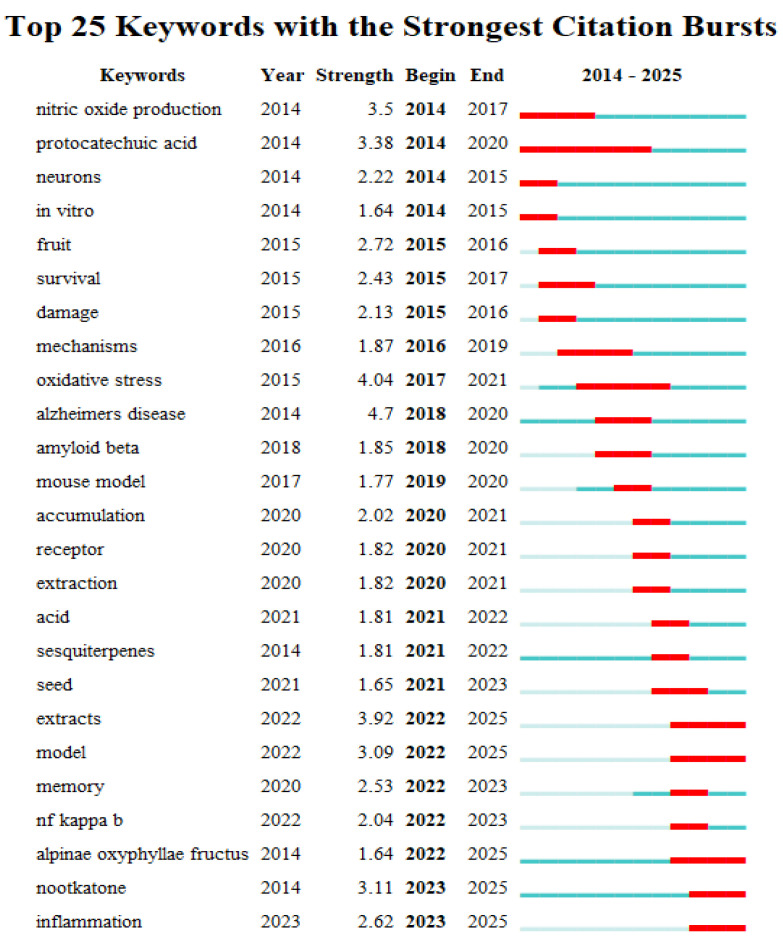
Top 25 burst keywords in English-language studies on *A. oxyphylla*.

Burst terms are keywords that exhibit significant fluctuations in frequency over specific time periods. Their distribution characteristics effectively reveal the dynamic evolution of academic research hotspots. In the keyword burst analysis plot, the blue line indicates the overall time interval, while the red line highlights periods during which a keyword shows a significant increase in usage. “Year” represents the earliest appearance of the keyword, “Intensity” indicates the burst strength, and “Start” and “End” denote the beginning and end of the burst period. The top 25 burst keywords were identified from the English-language literature (Figure 4). In the early stage, the terms with the highest burst intensity were nitric oxide production and protocatechuic acid. Emerging hotspots in the later stage included oxidative stress and Alzheimer’s disease. More recent focal terms include extracts, model, and nootkatone. Interestingly, research on *A. oxyphylla* demonstrates a clear developmental trajectory: beginning with in vitro investigations of extracts in the early phase, progressing to mechanistic studies in the intermediate phase, and, most recently, expanding to diverse biological pathways and molecular targets. Burst keyword patterns also reveal that research related to cognitive enhancement has remained consistently prominent across all stages. Current evidence suggests that, in Alzheimer’s disease, oxidative stress and Aβ accumulation act as initial triggers, activating NF-κB, the cell’s alarm system. Activated NF-κB then induces persistent neuroinflammation, ultimately contributing to neuronal injury and memory decline. Natural sesquiterpenes such as nootkatone are of particular interest because they may modulate multiple points along this pathological pathway. These compounds may exert direct antioxidant effects while simultaneously inhibiting NF-κB-mediated inflammation, thereby protecting neurons and enhancing cognitive function. This multi-targeted mechanism represents an important direction in current Alzheimer’s disease drug development.

In conclusion, research on the multi-target cognitive-enhancing effects of *A. oxyphylla* represents a promising direction for future studies. Mechanistic investigations of its extracts and associated bioactivities, particularly anti-inflammatory pathways, also hold significant potential for advancing the therapeutic development of *A. oxyphylla*-derived compounds.

## 4. Patents

**Figure 5:**
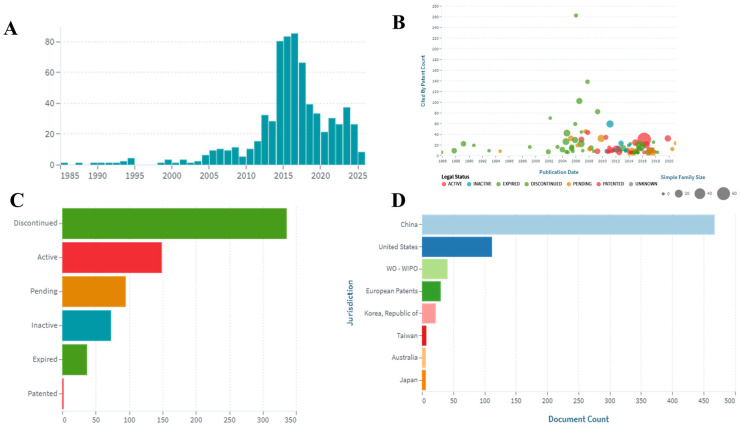
(**A**) Patent documents over time. (**B**) Citation distribution by publication date, legal status, and simple family size. (**C**) Legal status of patent documents. (**D**) Patent documents by jurisdiction.

Patent data were retrieved from the LENS database by searching “*Alpinia oxyphylla*” and “*Alpinia oxyphylla* miq” with the time span set from 1950 to 2025. Specific patent family classifications were obtained through the family grouping option, and all patent-related analytical data and visualization figures were generated from the analysis module. A search for *A. oxyphylla* identified 692 patents, consisting of 574 simple families and 118 extended families. Patent trends over time, top-cited patents, legal status, and jurisdictional distribution are shown in Figure 5A–D. In addition, the inventors of patent applications are presented in Figure S2 (Source: https://www.lens.org/lens/search/patent/analysis?q=Alpinia%20oxyphylla&P, (25 October 2025). Several patents focus on the essential oil components of *A. oxyphylla*. These components, together with Dendrobium, goji berry, wheat, soybeans, and other ingredients, are used as food additives. Cognitive-enhancing essential oil formulations can be used directly as flavoring agents or combined with different seasonings to improve the taste of food. Essential oils derived from high-altitude sources exhibit enhanced antioxidant properties, suggesting potential health benefits during daily consumption [21]. Another invention relates to the development of *A. oxyphylla* vinegar within the field of medicinal and edible health foods. The formulation contains *A. oxyphylla* fruit, longan, *Millettia speciosa* Champ., sea buckthorn fruit, maltose, brewed grain vinegar, sodium benzoate, sodium alginate, stevioside, caramel, citric acid, and sodium DL-malate. The processing method preserves the characteristic pharmacological effects of *A. oxyphylla*, including inhibition of ileum contraction and prostaglandin synthesis while enhancing its unique flavor [22].

## 5. Plant Morphology and Classification

**Figure 6:**
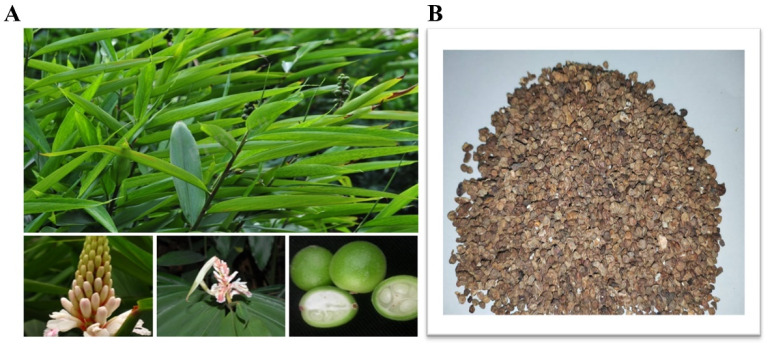
(**A**) Leaves, flowers, and fresh fruits of *A. oxyphylla* (source: https://www.iplant.cn/frps, accessed on 20 February 2026); (**B**) fruits of *A. oxyphylla*.

*A. oxyphylla*, commonly known as Yi Zhi Zi in China, is a perennial herb whose dried mature fruits, known as kernels, are widely used in traditional medicine. This species thrives in shaded, moist forests, with its native range extending across Hainan and parts of southern China [23]. The basic botanical characteristics of *A. oxyphylla* are summarized in Table 3. *A. oxyphylla* typically grows 1–3 m tall and exhibits a caespitose growth habit along its stems. The rhizomes are relatively short (3–5 cm). The leaf blades are lanceolate, and the racemes are enclosed within a hooded involucral bract during the bud stage. The flowers remain fully intact during anthesis, and the inflorescence rachis is sparsely covered with fine hairs. The fruit capsule is globose when fresh but becomes fusiform upon drying, featuring distinct vascular ridges on the pericarp and persistent calyx tube remnants at the apex [24]. The seeds are irregularly oblate and covered with a yellowish aril. Flowering occurs from March to May, while fruiting takes place from April to September. The fruits are harvested in summer and autumn as they ripen from green to red, then dried either in sunlight or at low temperatures. It is recognized as a locally crucial medicinal herb in Hainan and is regarded as one of the four primary southern medicines in China (https://www.iplant.cn/). Its morphological features are shown in Figure 6A,B.

## 6. Traditional Uses

According to the Chinese Pharmacopoeia (2020 edition), *A. oxyphylla* is defined as the dried mature fruit of the same plant, widely used in traditional Chinese medicine (TCM) for its therapeutic value (https://ydz.chp.org.cn/). *A. oxyphylla* is characterized by a warm nature and sweet taste, associated with the spleen and stomach meridians [25]. It is traditionally used to warm the spleen, stop diarrhea, and stimulate salivation. In TCM, it treats spleen coldness, diarrhea, abdominal pain due to cold, excessive salivation, and frequent urination [5]. The herb is also believed to stabilize semen and reduce urination. Typical dosage forms include decoctions (3–9 g), pills, and powders.

*A. oxyphylla* is also used in dietary therapies such as *A. oxyphylla* porridge, which is believed to tonify the kidney, warm the spleen, and alleviate diarrhea. However, it is contraindicated in individuals with yin deficiency (a TCM term referring to an insufficiency of the body’s cooling and nourishing energy) or internal heat (https://www.zgbk.com/). Classical TCM texts provide extensive documentation of their therapeutic roles. Kai Bao Ben Cao (开宝本草, 973–974 CE, Song dynasty, China) records the use of *A. oxyphylla* for spermatorrhea, enuresis, frequent urination, and qi (pronounced chee), referring to a lack of vital life energy in TCM theory, as well as for mental restlessness. It was believed to harmonize the upper and lower jiao (pronounced jee-ow; functional regions of the body in TCM) and regulate qi circulation. For nocturnal urination, 24 crushed fruits were decocted with salt. Ben Cao Bei Yao (本草备要, Wang Ang, 1694, Ming dynasty, China) notes the ability of *A. oxyphylla* to astringe essence, consolidate qi, warm the middle burner, promote salivation, aid digestion, and firm stools. It was used to treat vomiting, diarrhea, stomach cold, abdominal pain, and spermatorrhea.

Shi Yi De Xiao Fang (世医得效方, 14th century, Yuan dynasty, China) describes the Sanxian Pill, which used *A. oxyphylla* as a principal ingredient for nocturnal emission, vomiting, diarrhea, and abdominal cold pain. Guang Zhi (广志) cited *A. oxyphylla* for vomiting and retching, while Min Xue Qi Yuan (民学启源, ca. 18th century, Qing dynasty, China) emphasized its efficacy for spleen- and stomach-related disorders. Ben Cao Gang Mu (纲目, Li Shizhen, 1596 CE, Ming dynasty, China) lists the therapeutic applications of *A. oxyphylla* for treating abdominal pain, heart-qi deficiency, nocturnal emission, turbidity, fever, hemorrhage, and hematuria. Compound prescriptions also feature *A. oxyphylla* as a core component. Zhong Ding Yan Sheng Fang (重订严氏济生方, Yan Yonghe, ca. 1732 CE, Qing dynasty, China) prescribes a formulation combining *A. oxyphylla* (9 g) with dried ginger, roasted licorice, and fried fennel to treat kidney meridian obstruction, abdominal cold pain, and hernia-related discomfort. Jing Xiao Chan Bao (经效产宝, ca. 1820 CE, Qing dynasty, China) recommends a preparation containing *A. oxyphylla* (5 g) mixed with acceptable salt, ground into powder, and added to glutinous rice porridge to tonify the kidney, support yang (pronounced yahng; the active, warming energy in TCM theory), and reduce urination. This preparation has been used to treat menopausal syndrome and spleen and kidney yang deficiency, especially in elderly patients presenting with abdominal cold pain, frequent urination, and enuresis.

With the accumulation of empirical and clinical evidence, *A. oxyphylla* has been increasingly integrated into functional food applications. Modern products such as *A. oxyphylla* yogurt, tea bags, and solid beverages illustrate its growing relevance beyond traditional medicine.

### 6.1. Harvesting and Processing Methods

The harvesting and processing of *A. oxyphylla* have been documented since the Eastern Han Dynasty (25–220 CE), with early descriptions found in Yi Wu Zhi and Nan Fang Cao Mu Zhuang detailing its fruit morphology and flavor characteristics. During the Song Dynasty (960–1279 CE), texts such as Ben Cao Tu Jing provided further accounts of harvesting practices, while the Chinese Pharmacopoeia (2020) notes that collection typically occurs in summer and autumn. Recent studies [26,27] identified the optimal harvest time as about 100 days after flowering, when fruits show peak maturity and seed quality. Processing remains essential in traditional Chinese medicine, as it modifies chemical composition, enhances efficacy, and reduces toxicity [28].

### 6.2. Traditional Processing Methods

TCM emphasizes the importance of proper processing (Paozhi) to enhance the efficacy, safety, and usability of herbal medicines. Common steps include cleaning, impurity removal, slicing, and drying to improve purity and storage stability [29,30]. The concoction process, central to TCM preparation, involves frying, roasting, calcining, steaming, or boiling herbs to modify their medicinal properties, increase therapeutic potency, and reduce toxicity [31,32,33].

The earliest documented processing methods for *A. oxyphylla* are found in Lei Gong Bao Zhi Lun, an early classical text on herbal preparation, which describes procedures such as peeling and salt-frying. The Chinese Pharmacopoeia (2020 edition) specifies two main approaches: (1) impurity and shell removal followed by stir-frying in brine, or (2) pounding the material for use. Studies show that salt-processing enhances the pharmacological activity of various herbs. For instance, salt-roasted Phellodendron bark better regulates intestinal flora and improves yin-nourishing effects compared to raw material [34]. Similar mechanisms have been reported across salt-treated herbs, where synergistic drug-salt interactions improve therapeutic efficacy [35,36,37]. In TCM, *A. oxyphylla* is commonly pulverized and salt-roasted to enhance the bioactivity of its constituents and optimize clinical effectiveness.

### 6.3. Modern Processing Methods

Modern processing methods for *A. oxyphylla* integrate traditional knowledge with advanced pharmaceutical and food technologies to enhance bioactive components, therapeutic efficacy, and commercial value. The main modern approaches include: (a) Effervescent tablet formulation: Effervescent tablets represent a convenient and stable dosage form that preserves the plant’s bioactive compounds for therapeutic use [38,39]. (b) Optimization of the salt-burning process: Refining heating and salting conditions ensures maximum retention of volatile oils and other bioactive compounds, thereby maintaining the plant’s pharmacological potency [40,41]. (c) Improved decoction techniques: By optimizing temperature and soaking time, modern processing enhances the extraction efficiency of active ingredients while preserving their stability [42]. (d) HP-β-CD inclusion technology: The encapsulation of volatile oils with hydroxypropyl-β-cyclodextrin (HP-β-CD) improves their solubility, stability, and bioavailability in pharmaceutical applications [43,44]. While these techniques reflect significant progress, they remain rooted in traditional practices, blending heritage with modern scientific innovation. Processing regulations in provincial and municipal regions of China follow the Chinese Pharmacopoeia as the national standard, ensuring consistency across variations in soaking time, dosage, and other parameters.

Comparative studies between historical and modern methods reveal that technological advancements build upon empirical TCM knowledge to refine efficacy and standardization [45]. For instance, the Shanghai Specification for the Processing of TCM Decoction Pieces has been updated to incorporate modern insights. Studies show that the principal volatile oils, nocarone and eremophilene, remain stable during salt-processing, indicating that contemporary methods do not degrade these key pharmacologically active constituents [46].

Recent research also demonstrates that salt grilling effectively preserves the active components in nootropic kernels, thereby enhancing their pharmacological effects [47]. Salting alters the composition of volatile oils, water extracts, and nootkatone, which may contribute to the herb’s therapeutic efficacy [48]. Beyond traditional medicine, *A. oxyphylla* has gained popularity in the food industry. Processing techniques now support the production of candies, yogurts, beverages, and effervescent tablets, merging medicinal and culinary applications and expanding their commercial potential [47,49].

Collectively, these modern approaches provide a scientific foundation for improving processing precision, retaining active ingredients, and expanding the practical applications of *A. oxyphylla*, thereby reinforcing the connection between traditional wisdom and modern technology.

## 7. Phytochemicals

*A. oxyphylla* contains a broad spectrum of bioactive constituents responsible for its diverse pharmacological activities. Accordingly, the chemical composition of *A. oxyphylla* has been extensively investigated, as the fruit represents the primary medicinal part. The identified constituents belong to multiple structural classes, including terpenoids (both volatile and non-volatile), flavonoids, diphenylheptanoids, fatty acids, sterols, and polysaccharides.

### 7.1. Essential Oils

In TCM, volatile oils refer to a class of aromatic, water-distillable, and water-insoluble oily substances derived from plants. These oils are widely used in various fields, including food and medicine, due to their potential to combat drug-resistant pathogens [50]. The volatile oil fraction of *A. oxyphylla* contains numerous bioactive constituents, including terpenoids, phenolic compounds, and diphenylheptanoids [51,52]. Several studies have analyzed the volatile oil components of *A. oxyphylla* using advanced analytical techniques, including gas chromatography (GC), mass spectrometry (MS), supercritical CO_2_ extraction, high-speed countercurrent chromatography, and computer-assisted interpretation. Through supercritical CO_2_ fluid extraction, more than 200 compounds were identified in the volatile oil of the nootropic kernel [53]. Volatile oil extracted via steam distillation and analyzed by GC-MS revealed 122 chemical components, 42 of which had matching degrees above 85%, accounting for 74.35% of the total volatile oil content [54]. Among these compounds, p-cymene and nootkatone were present in considerable amounts [55]. The analysis revealed that p-cymene comprised 44.87% of the total volatile oil and exhibited mucodynamic, antitussive, and bacteriostatic properties [55]. Substantial evidence supports the antimicrobial, insecticidal, antioxidant, anti-inflammatory, anticancer, cardioprotective, neuroprotective, hepatoprotective, and nephroprotective activities of nootkatone [56,57].

The volatile oil content and composition vary among different fruit types of AOF and are influenced by processing conditions, such as drying. In addition to the fruit, the leaves of *A. oxyphylla* have also been reported to contain various volatile constituents [58]. Comparative analysis of the volatile oils from the fruit and leaves revealed that both share several major bioactive constituents. Particularly, 3-caren-10-aldehyde and 3-caren-10-alcohol were detected in the leaves but were absent in the fruit [59]. Using GC-MS coupled with steam distillation, 56 chemical components were identified in the volatile oil extracted from the leaves, representing 91.84% of the total oil content. Likewise, supercritical CO_2_ extraction identified 66 components, accounting for 98.71% of the oil. Both extraction methods revealed that terpenoids, including oxygenated terpenes, monoterpenes, and sesquiterpenes, dominate the volatile oil composition, with more than 85% of the components exhibiting strong pharmacological activity [60]. Although AOF represents the primary medicinal material, other tissues of *A. oxyphylla*, such as stems, leaves, and roots, remain relatively underutilized. To optimize resource use, volatile oils were extracted from different tissues of locally grown *A. oxyphylla* in Fujian using steam distillation. The volatile oil content was highest in the roots (0.22%), followed by the leaves and stems. A total of 52 compounds were identified, with olefins being the most abundant, followed by alcohols. Key constituents, including γ-terpinene, myrtaldehyde, α-pinene, β-pinene, β-basilene, and cymene, were detected in all tissues [61,62].

### 7.2. Terpenoids (1–112)

**Figure 7:**
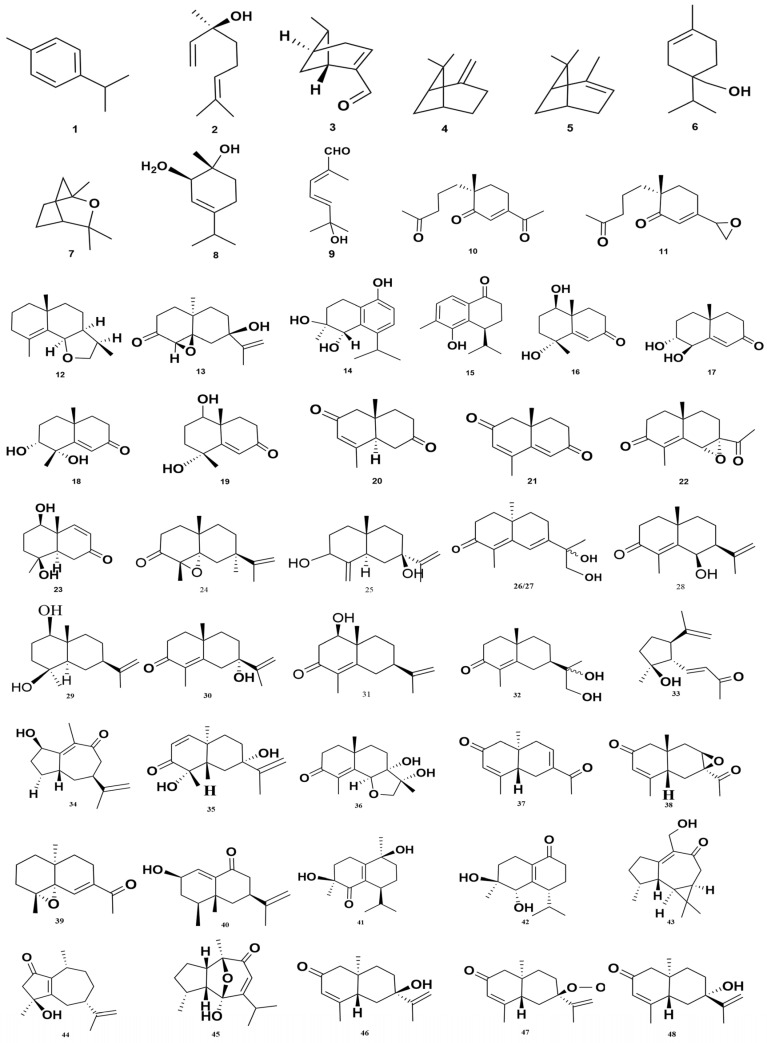

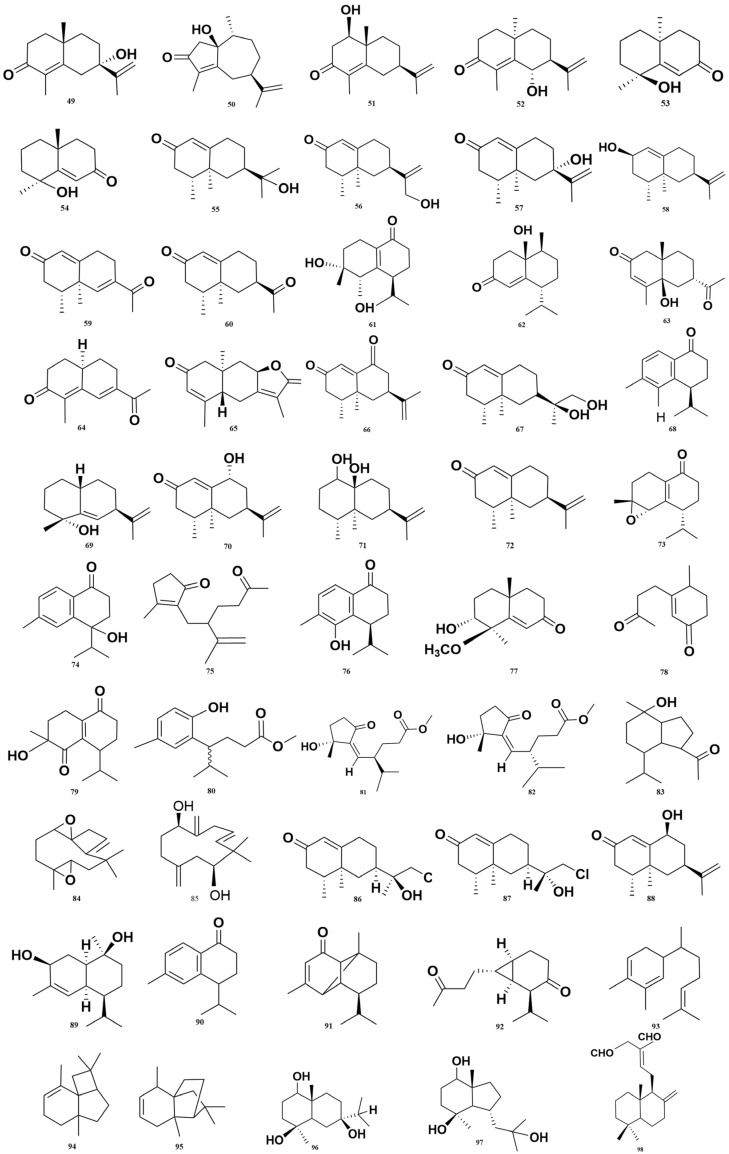

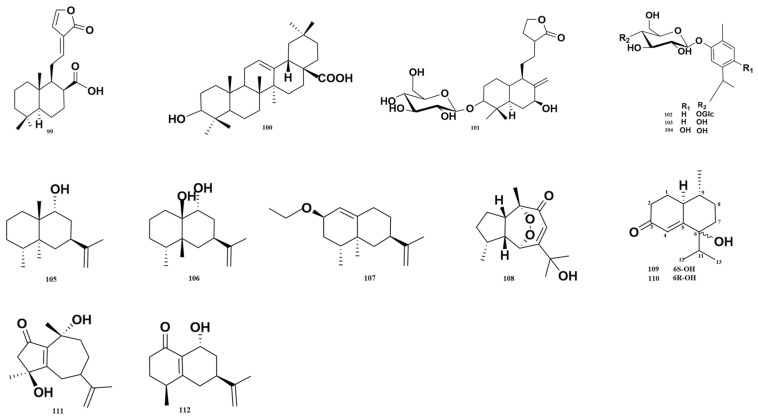
Structural details of various terpenes derived from *Alpiniae oxyphyllae* Fructus.

Although terpenoids constitute the principal components of the volatile oil fraction, they are discussed separately here due to their structural diversity and significant pharmacological relevance, including both volatile and non-volatile terpenoid derivatives. To date, approximately 112 terpenoids have been identified, predominantly sesquiterpenoids, along with monoterpenoids, diterpenoids, and triterpenoids. As shown in Figure 7, the major compounds include p-cymene (1), linalool (2), martial (3), β-pinene (4), α-pinene (5), terpinen-4-ol (6) [63], eucalyptol (7) [53], and (1R,2R)-p-menth-3-ene-1,2-diol (8) [64]. Other compounds include (2E,4E)-6-hydroxy-2,6-dimethyl-hepta-2,4-dienal (9) [65], oxyphyllones A–G (10–15) [66], oxyphyllone C (16) [67], oxyphyllenones A–C (17–19) [68,69], and oxyphyllanenes A–C (20–22) [70]. Further derivatives include 1β,4β-dihydroxy-11,12,13-trinor-8,9-eudesmen-7-one (23) [68] and oxyphyllanenes D–G (24–27). Compounds such as 5(4aS,7S,8R)-8-hydroxy-1,4a-dimethyl-7-(prop-1-en-2-yl)-4,4a,5,6,7,8-hexahydronaphtha-len-2(3H)-one (28), (±)1b,4b-dihydroxyeudesman-11-ene (29), and (4aS,7S)-7-hydroxy-1,4a-dimethyl-7-(prop-1-en-2-yl)-4,4a,5,6,7,8-hexahydronaphthalen-2(3H)-one (30) are also noted. Further terpenoids include ligucyperonol (31) and 7a(H),10b-eudesm-4-en-3-one-11,12-diol (32) [70]. Recent studies have reported oxyphyllins A–G (33–39), oxyphyllins H–J (40–42), aromadendr-1(10)-ene-9-one (43), (4S,7S,10R)-4-hydroxy-guaiane-1(5),11(12)-diene-2-one (44), alpineneone (45), teucrenone (46), and other eudesmane-type terpenoids such as (47–55) [9,71,72]. Additional compounds, including 12-hydroxynootkatone (56) [9], 7α-hydroxynootkatone (57), β-nootkatol (58), and oxyphyllanone A (59) [73], expand this chemical diversity. Other isolated terpenoids include oxyphyllenodiol A (62), (5R,7S,10S)-5-hydroxy-13-nore-udesma-3-en-2,11-dione (63), and (10R)-13-noreudesma-4,6-dien-3,11-dione (64), (5S, 8R, 10R)-2-oxoeudesma-3,7(11)-dien-12,8-olide (65), eremophila-1(10),11(12)-dien-2,9-dione (66), and (11S)-nootkatone-11,12-diol (67). Moreover, (4S)-10-nor-calamenen-10-one (68) [73], oxyphyllols A-B (69–70) [65], (+)-mandassidion (75) and (9E)-humulene-2,3,6,7-diepoxide (84) [74], a ingiberene (93), α-panasinsene, and γ-neoclovene (94–95) [53] have also been characterized.

**Figure 8:**
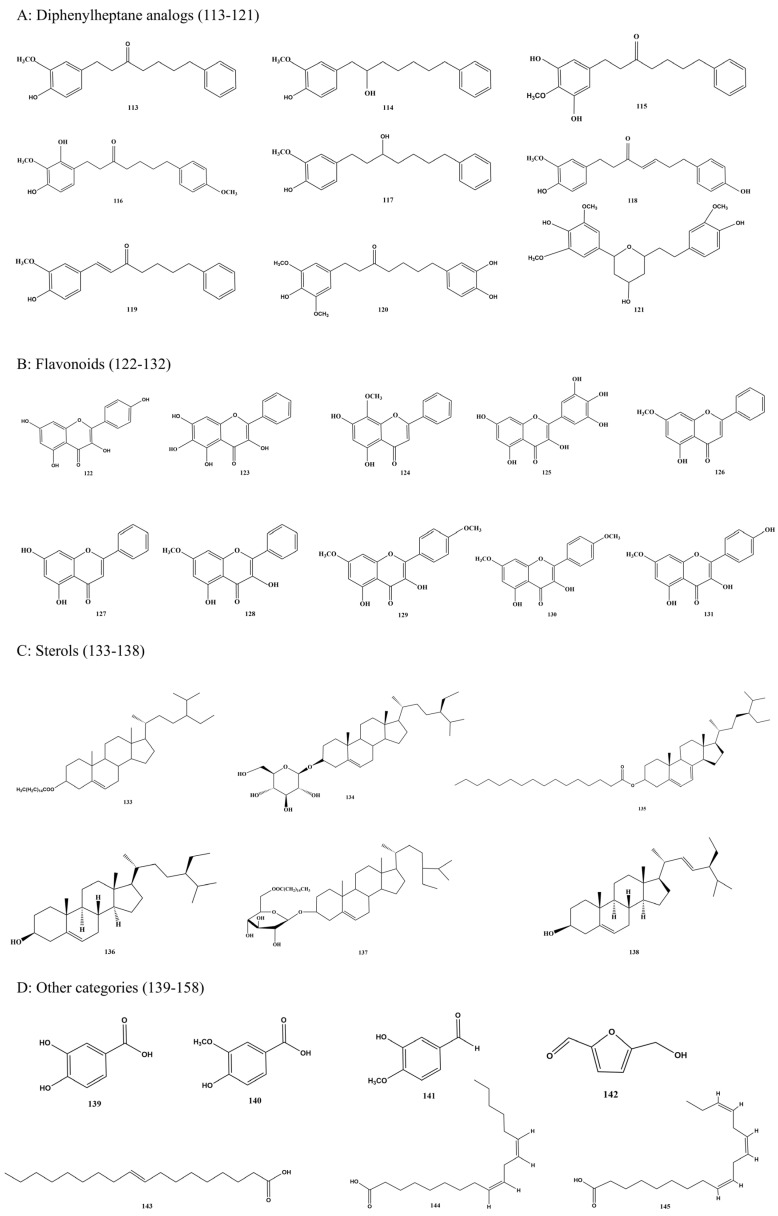
Structures of various bioactive compounds from *Alpiniae oxyphyllae* Fructus.

Further compounds such as 1β,4β,7β-trihydroxyeudesmane (96), bullatantriol (97) [75], (E)-labda-8(17),12-diene-15,16-dial (98) [71], (E)-labda-12,14-dien-15(16)-olide-17-oic acid (99) [76] and oleanolic acid (100) [77] exhibit significant pharmacological properties, especially anti-inflammatory and anticancer activities. Oxyphyllonesides A–B (101–102) [78] show antimicrobial potential, while carvacrol-2-O-β-glucopyranoside (103) and thymoquinol-2-O-β-glucopyranoside (104) [78] represent advances in natural product chemistry. Emerging compounds such as alpinoxyphyllols A-B (105–106), 2-O-ethyl-β-nootkatol (107), 11-hydroxyisohanalpinone (108), 6S-/6R-oxyphyllenone H (109–110) [79], cyperusol A (111), and (4S*,7S*,9R*)-14-nor-5(10),11(12)-dien-9-ol-1-one-eudesma (112) [80] further enrich the chemical diversity of *A. oxyphylla*. The structures of these compounds are illustrated in Figure 8.

Sesquiterpenoids from *A. oxyphylla* have been shown to enhance GLP-1 activity and exert multi-enzyme inhibitory effects through the Ca^2+^/CaMKII and PKA signaling pathways, suggesting potential for diabetes management [79]. HPLC analysis confirmed that nootkatone, a major sesquiterpenoid, protects against cerebral ischemia–reperfusion injury by modulating the p38 MAPK and JNK pathways [81]. The neuroprotective effects of *A. oxyphylla*, mediated by multi-target, low-toxicity mechanisms, particularly its sesquiterpenoids and monoterpenoids, have also been highlighted [82].

Further studies demonstrate that terpenoids in AOF inhibit MPTP-induced apoptosis in Parkinson’s models, upregulate dopaminergic neuron markers, and modulate metabolic pathways. Liu et al. found that nootkatone promotes glucose uptake, reduces hepatic triglyceride levels, and regulates AMPK and MAPK signaling, suggesting therapeutic potential for metabolic-associated fatty liver disease [83]. In neuroinflammation models, terpenoids significantly suppress M1 markers (NO, TNF-α, iNOS) and enhance M2 markers (IL-10, Arg-1), promoting an anti-inflammatory phenotype [84]. Overall, terpenoids derived from *A. oxyphylla* exhibit diverse pharmacological effects, including neuroprotective, anti-Alzheimer’s, anti-inflammatory, anticancer, and hepatorenal protective activities. For instance, nootkatone and tectochrysin jointly extend lifespan in C. elegans and activate insulin/IGF-1 and SKN-1 pathways, enhancing oxidative stress resistance [85].

### 7.3. Diphenylheptane Analogs (113–121)

**Figure 9:**
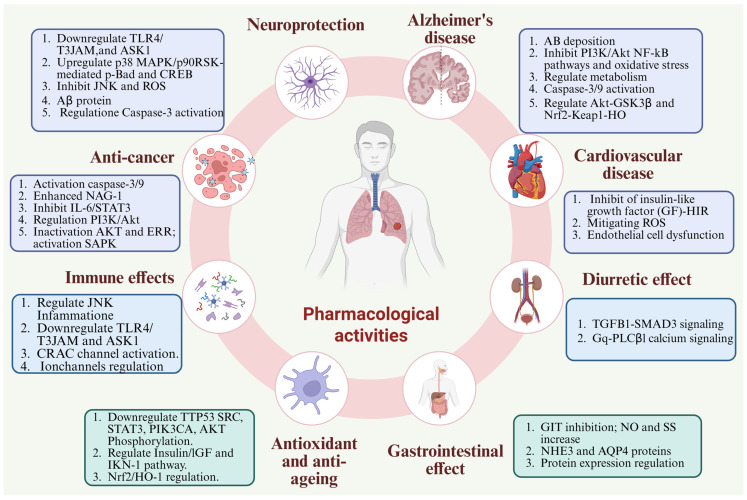
The figure highlights the complex therapeutic effects of *A. oxyphylla*, including its regulation of oxidative stress, immune responses, metabolic pathways, and key signaling mechanisms. These actions contribute to its potential in treating various conditions such as cancer, Alzheimer’s disease, cardiovascular diseases, and other chronic disorders.

Diphenylheptane analogs represent a primary class of phytochemicals in *A. oxyphylla*, with several members demonstrating significant antitumor potential. To date, nine such compounds have been isolated from this plant. The main compounds are shown in Figure 8, including yakuchinone A (113) [64,86], neonootkatol (114) [87], 1-(3′,5′-dihydroxy-4′-methoxyphenyl)-7-phenyl-3-heptanone (115) [75], 1-(2′,4′-dihydroxy-3′-methoxyphenyl)-7-(4′′-methoxyphenyl)-3-heptanone (116) [77], oxyphyllacinol (117) [86], (E)-1-(4′-hydroxy-3′-methoxyphenyl)-7-(4″-hydroxyphenyl)-hept-4-en-3-one (118) [73], yakuchinone B (119) [64,87], dihydrogingerenone B (120), and 1,5-epoxy-3-hydroxy-1-(4-hydroxy-3,5-dimethoxyphenyl)-7-(4-hydroxy-3-methoxyphenyl)-heptanes (121) [77]. The structural formulas of these compounds are shown in Figure 9.

Two known diarylheptanoid compounds were isolated from the ethyl acetate fraction of the fruit of *A. oxyphylla* (2015) [88]. Using in vitro assays on human umbilical vein endothelial cells and an in vivo zebrafish model, they assessed both the toxicity and anti-angiogenic activity of these compounds. The findings demonstrated that both compounds exhibit significant anti-angiogenic effects, suggesting they may contribute to the bioactivity of the ethyl acetate fraction. Similarly, new diarylheptanoid compounds were isolated from the fruit of *A. oxyphylla*, and their antioxidant potential was evaluated through systematic free radical scavenging assays. This work provides an essential foundation for developing *A. oxyphylla* fruit as a natural antioxidant resource.

### 7.4. Flavonoids (122–132)

Flavonoids are major bioactive constituents of *A. oxyphylla* and contribute substantially to its neuroprotective effects. To date, 11 flavonoids have been isolated. The main compounds are shown in Figure 8, including kaempferol (122), baicalein (123), wogonin (124), myricetin (125), tectochrysin (126), chrysin (127), izalpinin (128) [77], kaempferide (129) [75], kaempferol-7,4′-dimethylether (130) [75], rhamnocitrin (131) [69], and pinocembrin (132) [89]. Their structures are illustrated in Figure 9. Additional flavonoids identified in *A. oxyphylla* include baicalein, lyciumide, myricetin, and eight acetylated flavonol glucosides (aphnilides A–H) [88]. The diverse flavonoid constituents have also been shown to contribute significantly to the plant’s antioxidant activity [90]. Furthermore, chrysin, tectochrysin, and kaempferol were isolated from the ethyl acetate fraction, and chrysin was demonstrated to suppress TNF-α-induced inflammation by reducing IL-6 and IL-8 expression and inhibiting NF-κB pathway activation [91]. Flavonoids from *A. oxyphylla* have also been traditionally used to treat diarrhea, promote diuresis, and manage intestinal disorders [24]. Moreover, these flavonoids exhibit neuroprotective effects, improving memory and cognitive function by inhibiting NF-κB signaling and reducing inflammatory mediators [92].

### 7.5. Sterols (133–138)

Several sterol compounds have been identified from *A. oxyphylla*, as shown in Figure 8. The main compounds include β-sitosterol palmitate (133) [89], β-daucosterol (134), sitosterol palmitate (135), β-sitosterol (136) [93], daucosterol palmitate (137) [93,94], and stigmasterol (138) [93]. Their structures are shown in Figure 9. Other sterols detected include β-sitosterol, stigmasterol, and ergost-5-en-3-ol. The principal steroidal constituents of *A. oxyphylla* consist of β-sitosterol, daucosterol and its esters, and stigmasterol. These sterols exhibit diverse pharmacological activities, including neuroprotective, antioxidant, anti-inflammatory, and lipid-regulating effects, thereby contributing to *A. oxyphylla*’s therapeutic properties [95].

### 7.6. Other Categories (139–158)

In addition to the phytochemicals mentioned above, numerous other compounds have been isolated from *A. oxyphylla*. Figure 8 presents the main compounds, which specifically include protocatechuic acid (139) [89], vanillic acid (140), isovanillin (141), α-hydroxymethylfurfural (142) [94], oleic acid (143) [65], linoleic acid (144), linolenic acid (145) [71], asarone (146), 2S-pentanol-2-O-β-D-glucopyranoside (147) [89], palmitic acid (148), 4-methoxy-1,2-dihydrocyclobutabenzene (149) [96], 2S-butanol-2-O-β-D-glucopyranoside (150), and 1S-hydroxyethylbenzene-1-O-β-D-glucopyranoside (151), palmitoleic acid (152), lignoceric acid (153) [71], staphylionoside D (154), benzyl-1-O-β-D-glucopyranoside (155), 2-O-β-D-glucopyranosyl-(1S)-phenylethylene glycol (156), (S)-1-phenylethyl-β-D-glucopyranoside (157) [96], and 5-hydroxymethylfurfural (158) [97], etc. The structures are shown in Figure 9.

The volatile oils of *A. oxyphylla* contain valencene, nootkatone, α-panasinsene, intermedeol, zingiberone, eucalyptol, p-cymene, linalool, and myrtenal. These volatile constituents exhibit a wide range of pharmacological activities, including enhancing transdermal drug absorption, exerting neuroprotective effects, and demonstrating antibacterial properties. Fatty acid components, such as palmitoleic acid, oleic acid, linoleic acid, linolenic acid, lignoceric acid, and palmitic acid, also display antioxidant and anti-inflammatory potential. Moreover, phenolic acids such as protocatechuic acid and isovanillin have been reported to possess neuroprotective and antioxidant effects [98].

### 7.7. Bioactive Polysaccharides

Polysaccharides are complex carbohydrate macromolecules composed of monosaccharide units linked by glycosidic bonds, forming either linear or branched chains. In addition, due to their diverse structural characteristics and functional properties, polysaccharides have found broad applications in the pharmaceutical [99], environmental [100], and food industries [101]. They are widely distributed in nature, including plants, animals, and microorganisms, and typically possess hierarchical structural organization surrounding primary, secondary, tertiary, and quaternary levels [102,103]. Polysaccharides exhibit a broad range of pharmacological activities, such as antioxidant, anti-inflammatory, antitumor, hypoglycemic, and immunomodulatory effects [103]. Polysaccharides isolated from *A. oxyphylla* (AOFP) have been extracted using ultrasound-assisted enzymatic extraction [104], hot-water extraction [105], optimized extraction via response surface methodology [106], and aqueous alcoholic precipitation [107]. To date, six polysaccharides have been isolated, although some remain insufficiently characterized in terms of structure (Table 4).

The principal monosaccharides identified in *A. oxyphylla* polysaccharides include arabinose, galactose, glucose, xylose, mannose, galacturonic acid, and glucuronic acid, with glucose consistently detected across samples [107]. AOFP has demonstrated notable anti-inflammatory, antioxidant, and immunomodulatory activities [104,107].

## 8. Pharmacological Activities

*Alpiniae Oxyphyllae* Fructus (*A. oxyphylla*) is rich in various bioactive compounds and demonstrates multiple pharmacological effects, including neuroprotective, anti-diarrheal, anti-diuretic, and saliva-suppressing activities [85]. Historically, *A. oxyphylla* has been widely used in TCM to treat conditions such as intestinal disorders, urosis, spleen and stomach deficiency, cold, and as a tonic, aphrodisiac, and remedy for dementia [112,113]. Most pharmacological studies have investigated extracts, fractions, essential oils, or isolated compounds derived from the fruits of *A. oxyphylla*. Modern research highlights its antioxidant, anticancer, anti-inflammatory, anti-allergic, antimicrobial, and antidiabetic effects [85,114] (Figure 9).

### 8.1. Neurological Disorders

A substantial body of scientific evidence shows that *A. oxyphylla* contains various chemical compounds with neuroprotective properties. Damage to the nervous system, which plays a crucial role in regulating multiple physiological functions, can potentially lead to a wide range of neurological disorders. Complex mechanisms characterize these disorders and often lack effective clinical treatments. Several in vivo and in vitro studies have demonstrated that *A. oxyphylla* extracts possess the potential to promote neuroprotection through various mechanisms, including the regulation of metabolic disorders [9,115,116], antioxidant and anti-apoptotic effects [84,92], anti-neuroinflammatory properties [110,117], and promotion of nerve repair [118]. Furthermore, *A. oxyphylla* has demonstrated a therapeutic impact on various neurodegenerative diseases, most notably Alzheimer’s disease. Its mode of action is characterized by multi-target and multi-pathway interactions, coupled with a favorable toxicity profile [115,119,120].

#### 8.1.1. Neuroprotective Activity

A comprehensive analysis identified 49 bioactive phytochemicals in *A. oxyphylla* using ultra-performance liquid chromatography coupled with triple quadrupole mass spectrometry, in accordance with Lipinski’s five rules for drug-likeness. Among them, 26 target 168 key molecules involved in the treatment of neurodegenerative dementia. Additional compounds identified include herbone B, 5-hydroxy-1,7-bis(4-hydroxy-3-methoxyphenyl) heptaneptan-3-one (5-HYD), hydroxyethyldione, hydroxyethylpropanol, and butyl. The key phytochemicals in *A. oxyphylla* capable of modulating the pathogenesis of neurodegenerative dementia in a multi-targeted manner were identified as β-D pyranoside, chrysin, herbone A, rhamnetin, and rhamnoside [10]. Moreover, 35 sesquiterpene compounds were extracted from the *A. oxyphylla* kernel and evaluated for their neuroprotective effects in SH-SY5Y cells using H_2_O_2_ as a neurotoxin. Nine of these compounds showed significant neuroprotective effects, further demonstrating the potential of *A. oxyphylla* as a neuroprotective agent [9]. Apoptosis in the penumbra represents a key mechanism of cell death in the early stages of ischemia–reperfusion injury. Enhancing neuronal recovery in the penumbra could significantly aid stroke patients’ recovery [121,122]. Extracts of *A. oxyphylla* have been shown to exert beneficial effects on cerebral ischemic injury by downregulating JNK-mediated signaling in the peri-infarct cortex. The anti-infarct impact of the treatment was partially attributed to the JNK-mediated downregulation of TLR4/T3JAM and ASK1-related inflammatory signaling pathways in the penumbral cortex [123].

The extract demonstrated neuroprotective effects against mitochondria-associated apoptosis by inhibiting the release of cytochrome c (cyt c), Smac/DIABLO, and AIF from mitochondria into the cytoplasmic fraction. This anti-mitochondrial apoptotic activity was mediated through the upregulation of the p38 MAPK/p90RSK-dependent p-Bad and CREB signaling pathways, along with the downregulation of the JNK/histone B-dependent Bax and p53 pathways [81]. In addition, the blood–brain barrier permeability of the four major active components of *A. oxyphylla,* protocatechuic acid, chrysin, norcarone, and vitaxanthin was confirmed using a co-culture model comprising immortalized human brain microvascular endothelial cells (hCMEC/D3) and astrocytes (HA1800). The results indicated that protocatechuic acid exhibited high permeability [98]. Another study suggests that salicin demonstrates neuroprotective effects by inducing PC12 cells [124]. The findings showed that salicin enhances microglial polarization, specifically toward M1 and M2 phenotypes.

Additionally, salicin regulates neuroinflammation by triggering the expression of receptor 2 (TREM2) in myeloid cells through mechanisms involving the PI3K/AKT/GSK3β and BDNF/TrkB/TLR4 signaling pathways [85]. Additionally, the mechanism of action of protocatechuorellin in *A. oxyphylla* on rotenone-induced damage to PC12 cells has been suggested to involve the enhancement of cellular endogenous antioxidant enzymes, inhibition of reactive oxygen species (ROS) production, and activation of the Caspase-3 pathway [125]. Research also shows that *A. oxyphylla* extract regulates the MAPK (ERK1/2, JNK, and p38)/PA (uPA, tPA)/MMP (MMP2, MMP9) mediated regeneration and migration signaling pathways in Schwann cells. As a result, protocatechuic acid (PCA) plays a pivotal role in Schwann cell migration and the regeneration of damaged peripheral nerves [126]. Nootkatone, the principal active ingredient in *A. oxyphylla*, has been shown to have neuroprotective properties and beneficial effects in an Aβ1–42-induced mouse model of Alzheimer’s disease. Additionally, nootkatone mitigated LPS-induced learning and memory deficits in mice, an effect linked to attenuation of neuroinflammatory responses.

In conclusion, *A. oxyphylla* exhibits neuroprotective effects and can modulate the pathogenesis of neurodegenerative dementia through a multi-targeted approach. However, existing studies are limited, primarily focusing on cellular models, and the mechanisms underlying these processes remain poorly understood. Research has shown that the active compounds in *A. oxyphylla* can cross the blood–brain barrier. Therefore, further exploration of how these compounds interact with the blood–brain barrier could enhance the development of pharmaceutical treatments for neurological disorders.

#### 8.1.2. Alzheimer’s Disease

Alzheimer’s disease (AD) is a degenerative disorder of the central nervous system that primarily affects older adults, with onset typically occurring in late adulthood or pre-adolescence. The hallmark features of AD include progressive cognitive decline and behavioral impairments. The underlying mechanisms contributing to the pathogenesis of AD include the deposition of amyloid beta (Aβ) proteins, neurotransmitter dysfunctions, oxidative stress, free radical damage, and the involvement of immune responses, inflammation, and apoptosis in disease progression [114]. The mechanism of action of *A. oxyphylla* in treating AD is complex, involving multiple components, targets, and pathways. It has been demonstrated that the various components of the total *A. oxyphylla* extract can act synergistically to regulate metabolic disorders associated with AD, particularly those related to amino acid, lipid, and energy metabolism. *A. oxyphylla* may offer an effective therapeutic strategy for AD through its effects on fatty acid amide hydrolase (FAAH), peroxisome proliferator-activated receptor gamma (PPAR-γ), and acetylcholinesterase (AChE). Additional enzymes such as butyrylcholinesterase (BChE) and alcohol dehydrogenase 1C (ADH1C) are influenced by key compounds, including nootkatone, yizhiketone A, yizhiketone B, and yizhiketone C. Specifically, ADH1C has been shown to help mitigate AD progression and improve memory function through the action of 17 pharmacologically active substances, including nootkatone, yizhiketone A, yizhiketone B, izalpinin, and chrysin [127]. Sun et al. reported that the extract of *A. oxyphylla* regulates brain and plasma metabolites involved in amino acid, lipid, and energy metabolism, thereby improving memory and learning deficits in AD rats [115]. *A. oxyphylla* extract could ameliorate AD symptoms in mice by modulating basal metabolism and influencing metabolic pathways [116].

**Figure 10:**
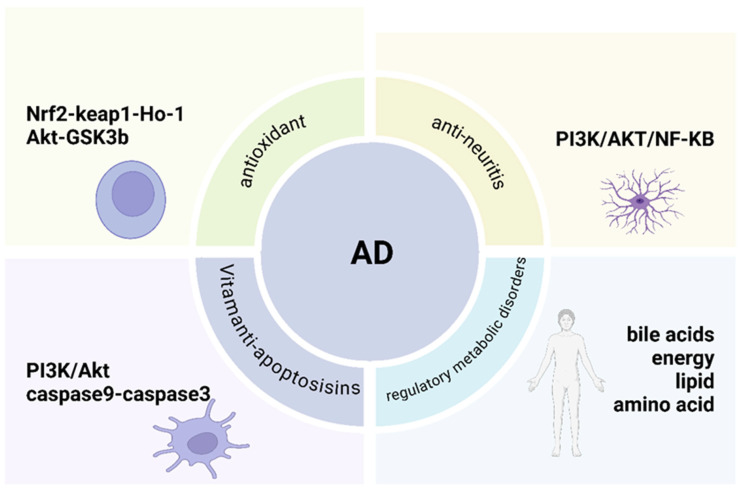
This figure illustrates the key molecular mechanisms by which *A. oxyphylla* exerts its therapeutic effects in AD, including the regulation of metabolic disorders, anti-inflammatory actions, antioxidant properties, and inhibition of apoptosis. These pathways collectively contribute to its neuroprotective effects and potential as a treatment for AD.

Additionally, research demonstrated that *A. oxyphylla* may inhibit oxidative stress-induced apoptosis through the PI3K/Akt pathway, providing a potential mechanism for AD treatment [128]. A novel compound extracted from *A. oxyphylla* has been found to reduce the expression levels of amyloid precursor protein (APP) and amyloid β-protein (Aβ), alleviating cognitive decline in SAMP8 mice. Furthermore, it exerts antioxidant effects via the Akt-GSK3β and Nrf2-Keap1-HO-1 pathways [129]. The research team led by He et al. [130] discovered that tectochrysin, a compound derived from an *A. oxyphylla* extract, has the potential to repair damage caused by Aβ1–42 in hippocampal CA1 neurons. This process is believed to involve regulating Aβ1–42 accumulation, modulating oxidative stress, and reducing total cholinesterase activity. A subsequent study found that Aβ triggers a series of apoptosis events by inducing an inflammatory response. However, the level of caspase-8 across groups did not change significantly, suggesting that ASHP may exert an anti-apoptotic effect not through caspase-8 but rather directly via caspase-9 to activate caspase-3 [131]. In 2020, a sesquiterpenoid compound, 2β-hydroxy-δ-cadinol (HOC), was isolated from *A. oxyphylla* fruits. The underlying molecular mechanisms of HOC in preventing Aβ1–42-induced neuronal apoptosis likely involve inhibiting Aβ1–42-induced ROS production, attenuating Aβ1–42-induced caspase-3 activation, and suppressing caspase-3 activity [132]. Research has also shown that *A. oxyphylla* alleviates cognitive deficits in an LPS-induced AD model by exerting anti-neuroinflammatory effects. This is achieved through the reduction in pro-inflammatory cytokines such as TNF-α, IL-6, and IL-1β via the PI3K/AKT/NF-κB signaling pathway [133]. Additionally, a heteropolysaccharide derived from *A. oxyphylla* (AOP70-2-1) has been found to exhibit potent anti-neuroinflammatory properties, significantly inhibiting the NF-κB pathway in the hippocampus of a mouse model [14]. The therapeutic potential of *A. oxyphylla* in treating AD lies in its ability to regulate metabolic disorders, along with its anti-inflammatory, antioxidant, and anti-apoptotic properties (Figure 10).

**Figure 11:**
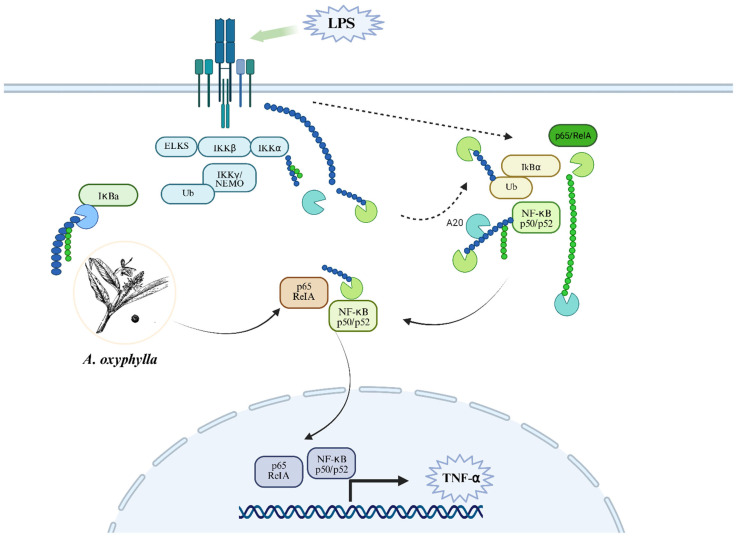
The activation of inflammatory pathways, leading to NF-κB signaling and NLRP3 inflammasome activation. These pathways drive the production of pro-inflammatory cytokines such as TNF-α and IL-1β, and compounds from *A. oxyphylla* are shown to inhibit these processes and reduce inflammation.

Among these, the anti-inflammatory, antioxidant, and anti-apoptotic effects of *A. oxyphylla* are particularly important. Growing research on metabolic regulation in AD treatment indicates that this area will become a key focus for exploring amino acid, lipid, energy, and bile acid metabolism in AD and other neurodegenerative diseases. The anti-inflammatory mechanism involves targeting key signaling pathways that regulate inflammation, thereby mitigating excessive immune responses and tissue damage. Overactivation of immune cells results in the release of inflammatory cytokines such as TNF-α, IL-1β, and INF-γ, driven by NF-κB, a central regulator of inflammation. Activated NF-κB promotes the transcription of pro-inflammatory cytokines (e.g., pro-IL-1β, pro-IL-18) [134]. Moreover, the NLRP3 inflammasome activates caspase-1, leading to the maturation of IL-1β and IL-18, which amplify inflammation [135]. Therapeutic strategies, such as natural compounds and targeted inhibitors, have shown the ability to suppress NF-κB and NLRP3 signaling pathways, reducing cytokine release and preventing excessive inflammation. These approaches highlight their potential to manage inflammatory diseases and maintain immune balance [136] (Figure 11).

### 8.2. Anti-Cancer Activity

**Figure 12:**
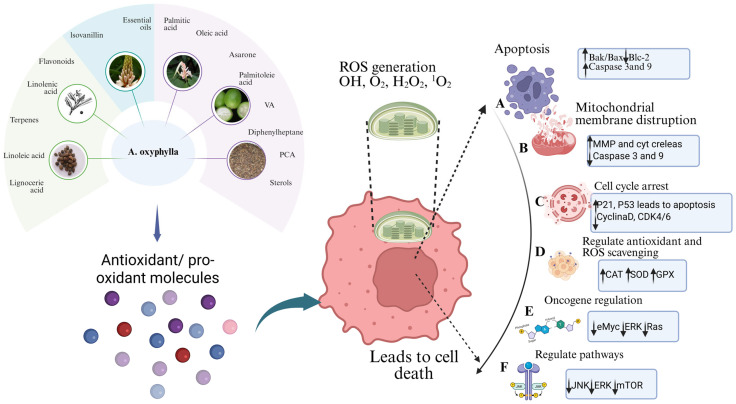
This figure highlights the anti-cancer potential of *A. oxyphylla*, emphasizing its bioactive compounds (e.g., essential oils, flavonoids, terpenes, sterols, diphenylheptane, and fatty acids) and their role in regulating oxidative stress. These mechanisms promote apoptosis, disrupt mitochondrial function, induce cell cycle arrest, and modulate oncogenes, contributing to its therapeutic effects against cancer. This concept is derived from the anticancer effects of phytochemicals, as outlined in [139].

The therapeutic potential of *A. oxyphylla* has been extensively explored, revealing significant anticancer properties across various cell lines. Studies have shown that different *A. oxyphylla* extracts significantly increased LDH release in HepG2 cells, indicating cellular damage. Additionally, the extract disrupted mitochondrial membrane potential and elevated intracellular ROS production, contributing to oxidative stress and cell death. This extract induced apoptosis in HepG2 cells by altering the Bax/Bcl-2 ratio, increasing cytosolic cytochrome c, and activating caspases 3 and 9. High ROS levels also led to DNA damage-mediated protein expression, inactivation of AKT and ERR, and activation of SAPKs. Moreover, it significantly inhibited HUVEC cell migration [136]. Nootkatone, an antitumor compound identified in *A. oxyphylla*, displayed antiproliferative and pro-apoptotic activities, as well as increased early growth response 1 (EGR-1) expression, which enhanced NAG-1 promoter activity [137]. Furthermore, petroleum ether extracts of *A. oxyphylla* have been shown to inhibit the growth of HepG2, BEL-7402, SMMC-7721, and Hep3B cells in a concentration and time-dependent manner, inducing apoptosis through the upregulation of PTEN expression, downregulation of PI3K expression, and inhibition of Akt phosphorylation, indicating a cancer-fighting mechanism via the PI3K/Akt pathway [138]. Phytochemicals present in *A. oxyphylla* exert anti-cancer effects by targeting key cellular processes in tumor cells. They combat oxidative stress by either neutralizing or generating ROS, which leads to cell death. Phytochemicals promote apoptosis by increasing proapoptotic proteins (Bak, Bax) and inhibiting antiapoptotic proteins (Bcl2), activating caspases (Caspase-3 and Caspase-9). They disrupt mitochondrial membrane potential, releasing cytochrome c and further triggering apoptosis. Phytochemicals arrest the cell cycle by upregulating tumor suppressor genes (p21, p53) and inhibiting cyclin-dependent kinases (CDK4, CDK6), preventing uncontrolled cell division. Additionally, they regulate oncogenes (c-Myc, ERK, Ras) to reduce tumor growth and upregulate antioxidant enzymes (CAT, GPX, SOD) to manage oxidative stress. Phytochemicals inhibit angiogenesis by downregulating Akt and blocking VEGF, reducing blood supply to tumors. They also suppress survival pathways (ERK, JNK, mTOR), limiting tumor cell proliferation. Together, these actions position phytochemicals as effective cancer treatment agents [139] (Figure 12).

### 8.3. Antioxidant and Anti-Aging Activities

*A. oxyphylla* is known for its culinary and medicinal properties, has been used in Chinese medicine for millennia, and is widely recognized as a dietary supplement. Its pharmacological activities include cardiotonic, neuroprotective, antioxidant, and renal and spleen-nourishing effects, which are associated with potential anti-aging benefits. Recent studies have explored the molecular mechanisms behind these effects. Using ultra-high-performance liquid chromatography coupled with quadrupole time-of-flight mass spectrometry (UHPLC-QTOF-MS), *A. oxyphylla* active constituents were identified and analyzed in conjunction with network pharmacology. The findings demonstrated that *A. oxyphylla* reduced levels of GLU, mAlb, Scr, BUN, MDA, and SOD while ameliorating renal pathological injury and cellular senescence in diabetic kidney disease (DKD) mouse models. In vitro experiments further revealed that serum containing *A. oxyphylla* increased HK-2 cell viability and mitigated cellular senescence under high-glucose conditions. Analysis identified 26 active compounds in the extract, and subsequent studies using network pharmacology, molecular docking, and Western blotting verified that *A. oxyphylla* exerts its effects by downregulating TP53 and phosphorylating SRC, STAT3, PIK3CA, and AKT [23]. Other studies have highlighted its benefits for improving egg quality and production by modulating reproductive hormones, antioxidant capacity, immunity, intestinal barrier function, and the cecum microbiota when used as a dietary supplement [5]. Concerning antioxidant activity, the ethanolic extract of *A. oxyphylla* and its principal components, proanthocyanidin B-2 and epicatechin, have demonstrated DPPH and ABTS free radical scavenging capabilities in both in vitro and shrimp feeding experiments. These compounds were shown to enhance the activities of antioxidant enzymes (CAT, GSH-Px, SOD, and T-AOC) and upregulate the expression of related genes (LvMn-SOD, LvCAT, LvproPo, and LvGSH-Px) in serum and liver [140]. Furthermore, polysaccharides (AOP) extracted from *A. oxyphylla* using hot water (AOP-HW), hydrochloric acid (AOP-AC), and NaOH/NaBH4 (AOP-AL) demonstrated efficacy in scavenging DPPH, hydroxyl, and ABTS radicals, with AOP-AL showing the highest activity [92]. *A. oxyphylla* has been shown to exert potent antioxidant effects through the Nrf2/HO-1 (heme oxygenase-1) pathway and free radical scavenging activity, with eudesma-3,11-dien-2-one and yakuchinone A identified as key active markers contributing to its antioxidant potential [75].

### 8.4. Anti-Inflammatory and Immune Activities

The inhibitory effects of monomeric compounds from *A. oxyphylla* on LPS- and IFN-γ-induced NO production in RAW264.7 murine macrophages were investigated. The study demonstrated that juniperane-type sesquiterpenes reduced NO release and confirmed that *A. oxyphylla* extracts possess anti-inflammatory and immunomodulatory properties [70]. The molecular mechanisms underlying the protective effects of *A. oxyphylla* extract in cerebral infarction were explored, focusing on the JNK-mediated inflammatory cascade in the penumbral cortex following transient middle cerebral artery occlusion (MCAo). The study revealed that *A. oxyphylla* extract treatment partially attenuated the inflammatory response by downregulating TLR4/T3JAM and ASK1-related signaling pathways in the penumbral cortex [123]. A novel acidic polysaccharide was isolated from *A. oxyphylla* that significantly inhibited LPS-stimulated NO release from BV-2 microglial cells and reduced the levels of IL-6 and TNF-α [134]. Moreover, the significant anti-inflammatory effects of nootkatone, oxyphyllenone A, and oxyphyllendiol A from *A. oxyphylla* were demonstrated by analyzing their ability to inhibit LPS-induced NO and TNF-α production in BV-2 microglial cells [63]. Recent studies have identified nootkatone’s potential to regulate calcium-signaling ion channels, particularly CRAC, K(V)1.3, and K(Ca)3.1, which are crucial for immune cell activation and proliferation. Electrophysiological studies in HEK293T cells overexpressing these channel proteins confirmed that nootkatone exerts a dose-dependent inhibition of channel currents without affecting cellular activity. Moreover, nootkatone treatment attenuated the inward calcium flux through activated CRAC channels, suggesting its role in regulating inflammatory T-cell activation [141].

### 8.5. Diuretic Activity

The ethyl acetate extract of *A. oxyphylla* seeds rapidly reduced urine volume in mice, with statistical significance (*p* < 0.05) in a urine-shrinking test, confirming the presence of active compounds with diuretic activity. Further isolation and identification revealed that 3-methoxy-4-hydroxy-diphenyhexane and 20-propyl-β-sterol were the key compounds responsible for *A. oxyphylla*’s diuretic effects [142]. The extract from *A. oxyphylla* seeds also exhibited a short-term antidiuretic effect 1 h after administration. UHPLC-ESI-QTOF/MS analysis identified three sesquiterpenoids, chlorophyll A, chlorophyll B, and nootkatone as the active components contributing to both the diuretic and antidiuretic effects [143]. In another study, the use of carbachol to induce detrusor muscle contraction in isolated rat bladders demonstrated that liangzhiin might be a potential lead compound for treating overactive bladder syndrome. Additionally, the treatment mechanism for overactive bladder (OAB) was linked to collagen synthesis via the TGFβ1-SMAD3 signaling pathway and the Gq-PLCβ1 calcium signaling pathway, both of which regulate urethral muscle contractions [25].

### 8.6. Gastrointestinal Protective Activity

The protective effects of the ethanolic extract of *A. oxyphylla* on castor oil-induced acute diarrhea in mice have been investigated. The study found that the extract significantly delayed the onset of diarrhea, reduced the proportion of wet feces, and inhibited intestinal motility. This effect was primarily attributed to inhibition of gastrointestinal transit (GIT), increased NO and somatostatin (SS) levels, and a reduction in histamine-induced spontaneous contraction of isolated guinea pig ileum. The analysis identified diphenylheptanoids, sesquiterpenoids, and flavonoids as the main active compounds responsible for these medicinal effects [144]. Additionally, the bioactive components used to treat diarrhea were explored. The study demonstrated that poplar bud xanthophylls, nootkatone, and puerarinone A have antidiarrheal effects by modulating NHE3 and AQP4 proteins via the action of poplar bud flavin [143,145].

### 8.7. Cardiovascular Protective Activity

A rat aging model was developed using D-galactose, and the crude *A. oxyphylla* extract obtained by solvent extraction was used to intervene in this model. The results demonstrated that the crude extract protected against cardiac hypertrophy. It was further observed that *A. oxyphylla* also played a protective role in reducing ventricular hypertrophy, primarily by modulating specific signaling mechanisms, providing experimental support for its potential cardiovascular protective effects [146,147]. Additionally, the therapeutic efficacy of *A. oxyphylla* in treating Ang II-induced cardiac hypertrophy was evaluated. The findings revealed that *A. oxyphylla* could inhibit the growth of insulin-like growth factor (IGF)-II/IIR by specifically suppressing its growth in H9c2 cells, thereby protecting against Ang II-induced pathological hypertrophy through the IGF-II/IIR-related signaling pathway [148].

### 8.8. Other Activities

A diabetic model was simulated, demonstrating that, by regulating intestinal flora composition, treatment with *A. oxyphylla* extracts lowered blood glucose levels and significantly reduced kidney damage in T2DM mice [149]. The ethanolic extract of *A. oxyphylla* and its dichloromethane crude fraction exhibited protective effects against CCl4-induced liver injury both in vivo and in vitro, significantly reducing alanine aminotransferase, alkaline phosphatase, serum aspartate aminotransferase, and total bilirubin levels in the model group. The NF-E2-related factor (Nrf2) pathway played a key role in mitigating oxidative stress [113]. Furthermore, the volatile oil extract of *A. oxyphylla* significantly enhanced the transdermal absorption of drugs without causing skin irritation or toxicity [150,151]. *A. oxyphylla* also contains various sesquiterpenoids, whose antidiabetic efficacy is primarily attributed to the stimulation of GLP-1 secretion and inhibition of α-glucosidase and PTP1B [79].

## 9. Conclusions

*A. oxyphylla* remains a high-value medicinal and edible resource that continues to attract increasing scientific and industrial interest. Recent phytochemical evidence indicates that the chemical space is still expanding. Beyond the abundant volatile constituents dominated by terpenoids (especially sesquiterpenoids), new terpenes, diarylheptanoids, flavonoids, phenolic acids, and structurally diverse polysaccharides have continued to be reported since 2018. This reinforces *A. oxyphylla* as a chemically rich system that may support multi-target bioactivity while also underscoring the need for stronger standardization when comparing results across laboratories and regions. Across pharmacological domains, the most consistent preclinical evidence supports antioxidant and anti-inflammatory activities with potential relevance to neuroprotection, gastrointestinal protection, and kidney-related benefits. Neurodegeneration, particularly AD, continues to dominate *A. oxyphylla* research, but the field is shifting from descriptive “extract activity” toward exposure-aware and mechanism-anchored studies. This transition is scientifically necessary: CNS claims require evidence that bioactive constituents (or their metabolites) reach brain-relevant compartments and engage plausible therapeutic nodes rather than relying solely on peripheral biomarkers. New analytical pharmacological workflows now provide a clear template for future CNS-focused research, integrating high-resolution profiling with sequential metabolism experiments to prioritize brain-accessible constituents and inform dose rationale. Our bibliometric and patent analyses align with this maturation trajectory. Publication growth has accelerated since 2020, and patent activity indicates active commercialization in essential-oil formulations, functional foods, and cognition-oriented products. These translational indicators are promising; however, they also highlight the importance of reproducible quality control, safety evaluation, and clinically meaningful endpoints to ensure that innovation remains aligned with robust mechanistic and clinical evidence.

## 10. Perspectives

Several challenges currently limit the global credibility and translation of *A. oxyphylla* research. First, terminology and material definition remain inconsistent in the literature: the plant species is sometimes conflated with the pharmacopeial drug (AOF) and with processed “kernels/seeds.” Future work should explicitly specify whether experiments used whole dried fruits, dehulled kernels, or defined fractions, and should authenticate materials with voucher specimens and standardized reporting. Second, quality control must address geo-herbalism and processing variability. Region-, genotype-, and processing-linked chemical differences can be significant enough to shift apparent bioactivity; therefore, multi-marker quality control strategies should replace single-marker approaches and should be linked to metabolomic fingerprints and validated marker panels. Third, mechanistic claims should increasingly move beyond general oxidative-stress and NF-κB narratives toward causal validation, including target engagement, pathway agitation, and synergy/antagonism testing for multi-component preparations. Fourth, pharmacokinetics, metabolism, and blood–brain barrier (BBB) penetration should become core requirements for CNS indications. Integrating absorption, distribution, metabolism, and excretion (ADME) and biotransformation profiling with efficacy models will help distinguish compounds that are mechanistically plausible in vivo from those that are active only under in vitro exposure conditions. Fifth, safety evidence remains uneven across extracts, fractions, and isolated constituents. Standardized *A. oxyphylla* products intended for long-term use in functional foods or supplements will require regulatory-grade safety packages, including an evaluation of potential interactions with vulnerable populations. Finally, clinical evidence remains limited relative to preclinical findings for most indications. The next phase should prioritize well-designed human studies for a small number of evidence-supported indications, using standardized materials, transparent dose rationale, and biomarker-linked endpoints.

## Figures and Tables

**Table 1:** Top 9 countries ranked by number of publications.

Rank	Countries	Number of Publications
1	People’s Republic of China	186
2	South Korea	19
3	USA	13
4	India	6
5	Japan	5
6	Vietnam	3
7	Turkey	2
8	Australia	2
9	Mexico	2

**Table 2:** Top 19 keywords in the English literature related to *A. oxyphylla*.

Label	Count	Centrality	Keywords
1	117	0.31	*Alpinia oxyphylla* Miq.
2	28	0.14	Oxidative stress
3	26	0.16	Protocatechuic acid
4	23	0.06	in vitro
5	20	0.06	Alzheimer’s disease
6	16	0.17	Apoptosis
7	16	0.09	Mouse model
8	16	0.07	Constituents
9	15	0.05	Nitric oxide production
10	14	0.06	Fructus
11	14	0.1	Cells
12	12	0.1	Nootkatone
13	10	0.03	Extracts
14	10	0.05	Antioxidant
15	10	0.15	Activation
16	10	0.04	Absolute stereostructures
17	9	0.01	Antioxidant activity
18	8	0.01	Essential oil
19	8	0.08	Accumulation

**Table 3:** Taxonomic classification of *A. oxyphylla*.

Scientific name: *Alpinia Oxyphylla* Miq. Common names: Yizhi Ren, Yizhi Zi, Zhaiding Zi Kingdom: Plantae Phylum: Streptophyta Class: Equisetopsida Subclass: Magnoliidae Order: Zingiberales Family: Zingiberaceae Genus: Alpinia Species: *Alpinia oxyphylla*

Cited from: Plants of the World Online (POWO). Kew Science. https://powo.science.kew.org/.

**Table 4:** Bioactive polysaccharides in AOF.

Name	Relative Molecular Mass (kDa)	Monosaccharide Composition	Chemical Structures	Biological Activities	References
AOFP1	43.4	Ara, Gal, Glc, Xyl, Man, GalA, and GlcA = 16.46:12.7:4.9:17.11:4.35:6.52:6	(1 → 4)-β-D-Xylp, (1 → 3,5)-α-L-Araf	Immunomodulatory activity	[107]
AOFP2	43.4	Glc	T-α-Glcp, (1 → 4)-α-D-Glcp-(1 →, and (1 → 4,6)-α-D-Glcp-(1 →	Immunomodulatory activity	[108]
AOFP3	44.3	GalA:Glc = 22.46:77.54	T-Glcp, (1 → 4)-GalAp-(1 →, (1 → 4)-Glcp-(1 →, and (1 → 4,6)-Glcp-(1 →	Immunomodulatory activity	[107,109]
AOP-AC	121.28	Glc = 94.77	−	Antioxidant and α-amylase inhibitory activities	[92]
AOP-HW	385.42	Glc = 52.31	−	Antioxidant and α-amylase inhibitory activities	[92]
AOP-AL	232.40	Ara, GalA, Gal = 28.42:17.61:17.09	−	Antioxidant and α-amylase inhibitory activities	[92]
AOP70–2-1	76.66	Man:Rha:GlcA:Glc:Gal:Xyl:Ara = 2.60:1.95:6.73:1.81:21.08:40.59:43.83	D-Glcp-(1→, (1 → 2,3,6)-DGalp-(1→, L-Araf-(1→, (1 → 2,5)-L-Araf-(1→, (1 → 4)-D-Glcp-(1→, (1 → 3,4)-DXylp-(1→, (1 → 3,6)-D-Manp-(1→, and L-Rhap-(1→, with a ratio of 11: 6:12:7:1:4:5:5	Anti-neuroinflammation	[14]
AOP70–1	5.3	Man:Rha:GlcA:Glc:Gal:Xyl:Ara = 5.40:2.20:3.70:3.10:11.10:30.00:44.60	L-Araf-(1→, (1 → 5)-L-Araf-(1→, (1 → 2,4)-D-Xylp-(1→, (1 → 2,3,4)-D-Xylp-(1→, D-Xylp-(1→, L-Rhap-(1→, D-Manp-(1→, (1 → 4)-D-Glcp-(1→, (1 → 6)-D-Glcp-(1→, D-Galp-(1→, (1 → 2)-D-Galp-(1→, (1 → 3,6)-D-Manp-(1 →, and (1 → 4)-D-GlcAp-(1 →, with a ratio of 4:7:6:2:1:1:4:1:2: 2:1:2:2	Anti-neuroinflammation	[110]
AOP	1151.9	Man:Rha:GlcA:GalA:Glc: Gal:Xyl:Ara = 0.71:2.05:2.41:31.39:33.31: 8.54:10.50:11.10	−	Anti-renal injury	[111]

Abbreviations: Ara, Arabinose; Gal, Galactose; Glc, Glucose; Xyl, Xylose; Man, Mannose; GalA, Galacturonic acid; GlcA, Glucuronic acid, −; not studied.

## Data Availability

The original contributions presented in this study are included in the article/Supplementary Materials. Further inquiries can be directed to the corresponding authors.

## Supplementary Materials

The following supporting information can be downloaded at: https://www.mdpi.com/article/10.3390/foods15071212/s1, Figure S1: The co-occurrence network map of major contributing countries; Figure S2: Top applicants of *A. oxyphylla* patent documents, illustrating the leading organizations and their respective levels of patent activity.
