# Supplementary material for: Phytochemical Diversity, Mechanistic Pharmacology, and Therapeutic Potential of Alpinia oxyphylla
Source: Foods. 2026 Apr 2;15(7):1212. doi: 10.3390/foods15071212 (PMC13074042; doi:10.3390/foods15071212)
Supplement: Supplementary file 1 [file foods-15-01212-s001.zip › foods-4191153-supplementary.pdf]

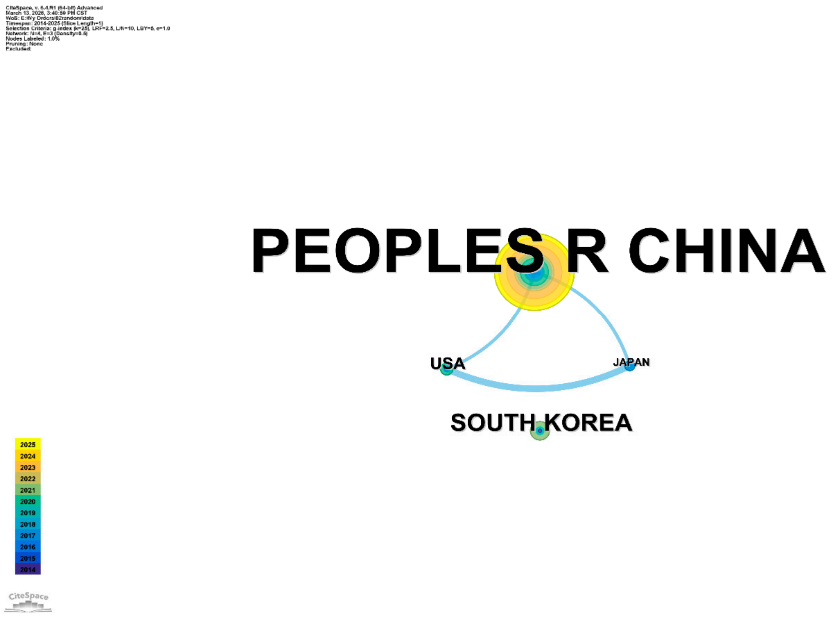

Figure S1. The co-occurrence network map of major contributing countries.

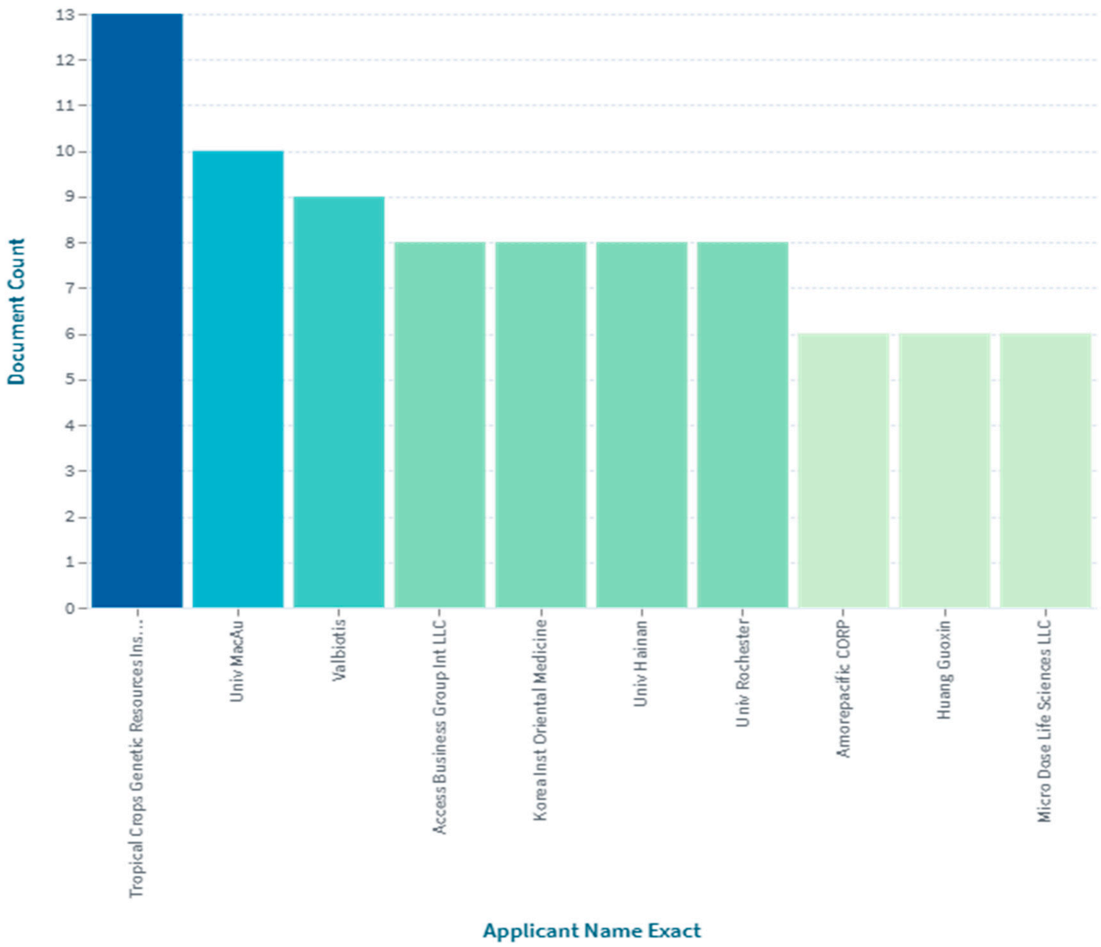

Figure S2 Top applicants of *A. oxyphylla* patent documents, illustrating the leading organizations and their respective levels of patent activity.
